# Community acceptance and willingness to pay for hypothetical COVID-19 vaccines in a developing country: a web-based nationwide study in Nigeria

**DOI:** 10.11604/pamj.2021.40.112.27780

**Published:** 2021-10-21

**Authors:** Ukamaka Gladys Okafor, Abdulmuminu Isah, Jude Chidiebere Onuh, Chiagozie Bonita Mgbemena, Chukwuemeka Michael Ubaka

**Affiliations:** 1 Pharmacists Council of Nigeria, Abuja, Nigeria,; 2 Department of Clinical Pharmacy, Pharmacy Management, University of Nigeria, Nsukka, Enugu State, Nigeria,; 3 Mobys Healthcare Limited, Abuja, Nigeria,; 4 Department of Prevention Care and Treatment, Institute of Human Virology, Abuja, Nigeria

**Keywords:** COVID-19, hesitancy, vaccine, willingness to accept, willingness to pay

## Abstract

**Introduction:**

some promising COVID-19 vaccines are soon to be available but getting the African community to accept them may be challenging. This study assessed the acceptability and willingness to pay (WTP) for hypothetical COVID-19 vaccines among Nigerians.

**Methods:**

a cross-sectional, web-based study was conducted among the Nigerian populace. A 20-item questionnaire was used to collect responses through Google form which was shared to consenting participants through two social media platforms. Multivariate logistic regression was used to determine the sociodemographic factors that were predictive of respondents´ willingness to accept the COVID-19 vaccines. Statistical significance was set at p<0.05.

**Results:**

six hundred and eighty-nine respondents completed the survey, with 50.5% being females. Exactly 43.3% of respondents reported that they would accept a hypothetical vaccine if it is currently available, 62.1% said they would accept it in the future while 71.1% agreed to accept it if recommended by healthcare providers. A third (31.9%) of respondents accepted the vaccine for their self-protection and half of those not accepting it (51.3%) said they did not want to “be used as an experiment”. Respondents who were of oldest ages (aOR=0.330, 95% CI: 0.141-0.767, p=0.010), of Christian religion (aOR=3.251, 95% CI: 1.301-8.093, p=0.011), and aware of a possible vaccine being made available (aOR=0.636, 95% CI: 0.440-0.920) were significantly more unwilling to accept the vaccine. The median range of WTP was US$1.2-2.5.

**Conclusion:**

there is a low acceptance in Nigeria for a COVID-19 vaccine if it was available now, but much higher if it is recommended by a healthcare provider. A high proportion of willing respondents indicated a positive WTP for the vaccine.

## Introduction

As at November 29^th^, 2020, the global burden of the novel corona virus disease (COVID-19) pandemic was reported by the World Health Organization to include 61,869,330 million confirmed cases, from which 1,448,896 deaths had resulted [1]. The pandemic has strained the health sectors of both the developed and developing nations, such that, with a definite cure not in sight, non-pharmacological preventive measures have been the most advocated strategies in public health promotion campaigns worldwide. The condition is worse in the low- and middle-income countries where even the healthcare workers are grossly underprepared to provide COVID-19 health education to the populace [2]. The pharmacological strategy with greater hope at the moment is the use of vaccines to confer immunity on the populace. However, the challenge that health professionals, policymakers and political leaders envisage is community's willingness to be vaccinated once an approved vaccine is available. As the world awaits this vaccine, it is good to remember that vaccinations stop pandemics, not vaccines. It has been reported that many Americans, at least one in three, would be reluctant to subject themselves to be vaccinated, notwithstanding the recommendation of the food and drug administration (FDA) and even if the vaccines were to be provided at no cost to them [3].

In Africa, majority of Nigerians would be unwilling to take an approved COVID-19 vaccine, but have been reported to be unwilling to submit themselves for any trial towards the approval of the vaccine [4]. The availability and mass distribution of a COVID-19 vaccine which can be considered to be safe is a global public health necessity if this pandemic is to be controlled [5]. High incidence of infectious diseases has been recorded in places where people declined or resisted vaccination [6]. For instance, the Northeast of Nigeria was severely plagued with polio virus until recently due to public resistance and outright rejection of the polio vaccine by the people [7,8]. Very little is known about the acceptability of a COVID-19 vaccine and the factors that inform the choices of the people about such a vaccine. This study is necessitated by the paucity of data on the acceptability and willingness of Nigerians to take the COVID-19 vaccine. A previous study by Enitan (2020) [3] reported that 80% of its respondents were unwilling to participate in a COVID-19 vaccine trial. Therefore, this study was conducted to evaluate the acceptability and willingness to pay for a hypothetical vaccine that is effective against COVID-19 among Nigerian citizens.

## Methods

**Study design:** a cross-sectional study design was adopted in this study. Responses were collected from eligible respondents using a 20-item questionnaire that was administered through web-based media between October 18^th^ and October 30^th^ 2020.

**Study area and study population:** the study was conducted in the Federal Republic of Nigeria, a country with a population of 200 million people spread across two major regions; Northern and Southern regions. Nigeria has had a past history of epidemics including Yellow fever, influenza, Lassa fever, Ebola, and is presently experiencing the COVID-19 outbreak like other countries in sub-Saharan Africa.

**Eligibility criteria and study sample:** only adult Nigerians (defined in the country as persons that are aged 18 years and above and eligibility was sought) who had access to internet services, irrespective of gender and cultural background in all the zones were invited through social media platforms to participate in the survey. Considering the estimation that about half of the population is aged 18 years and above, the sample size was calculated using Raosoft online sample size calculator (with 5% margin of error, 95% confidence interval and response distribution of 50%) to be 385. The desired sample size was increased by twice its size (770) in order to cover all the zones of the country as much as possible.

**Instrument for data collection:** the instrument used for data collection was purposely designed for the study. Literature search was conducted in physical and online databases to draft questions that were considered to measure the study objective. The questions were written in clear, simple, and unambiguous terms for easy comprehension. The first part of the instrument assessed respondents´ socio-demographic characteristics, including age, gender, marital and educational status. History of test for COVID-19 was also assessed in this part. Three closed-ended questions (scenarios) were asked respondents on their willingness to get vaccinated: 1) now if a vaccine was made available; 2) sometime in the near future and; 3) if their healthcare provider recommended, they get vaccinated. In the latter part of the instrument, the respondents willing to receive vaccination were asked two open-ended questions on willingness to pay for the vaccine and how much to pay, if the vaccine was not free of charge. The questions were subjected to content validation by inviting 10 randomly selected members of staff from different faculties at the University of Nigeria. Thereafter, 20 social media users were requested to go through the questionnaire to determine the face validity. The inputs from the validation processes were considered in preparing the final questionnaire, which was used in data collection. The key outcomes of the study were willingness to accept the COVID-19 vaccine and potential amount to pay for one if it is not provided free.

**Method of data collection:** the questionnaire was converted into a Google form and was shared to participants from the six geopolitical zones of the country through two popular social media and messaging platforms, WhatsApp and Facebook, requesting interested individuals to click on the web-link to complete the question. On clicking the web-link, an introductory interface first appeared where the study participants see the title of the survey, aim of the study and provide consent to participate in the study. The data was automatically retrieved from the Google database at the end of the response collection period.

**Data analysis:** the downloaded data were entered using Microsoft Excel (2019) and cleaned and there were no missing data as the form forced respondents to make a choice for closed questions. The data was exported into IBM Statistical Product and Services Solutions (version 25). Descriptive statistics were carried out to measure frequencies and percentages of the variables. Beyond descriptive statistics, bivariate analysis was conducted using chi-square and multivariate logistic regression (while including all independent variables in the model) to identify predictors of readiness of Nigerians to participate in COVID-19 vaccination. Statistical significance was set by p < 0.05.

**Ethical consideration:** this study did not directly involve sick patients of a particular disease and was considered institutional review board (IRB) exempt. However, an ethical clearance was granted by University of Nigeria Teaching Health Research Ethics Committee (Approval Number; NHREC/05/01/2008B-FEW00002458-1RB0002332). Informed consent was obtained from the participants with assurance of anonymity and confidentiality prior to their participation in the survey. Respondents were given the right to refuse to take part in the survey. The participation in the survey was made voluntary, without any form of coercion and was not compensated. Anonymity and confidentiality were maintained throughout the study. All data were stored in a protected file with access to information limited only to the researchers in charge of the survey.

## Results

**Sociodemographic characteristics:** a total of 689 respondents out of the 770 who accessed the form completed the survey (participation rate of 89.5%). There was an almost equal distribution of the respondents based on gender, with 348 (50.5%) being females, while 342 (50.2%) were married and staying with their spouses. The age category that was most represented in the study was 25-34 years: 208 (30.2%) (Table 1).

**Table 1:** sociodemographic characteristics of the respondents and their willingness to accept COVID-19 vaccines

Variable	Total	Accept vaccination now	Accept vaccination in near future	Accept vaccination if recommended
	**n (%) willing**			
All participants	689	298 (43.3)	426 (62.1)	486 (71.1)
Gender		0.109	0.400	0.294
Female	348 (50.5)	142 (40.8)	214 (61.5)	240 (70.0)
Male	341 (49.5)	185 (45.7)	212 (62.7)	246 (72.1)
Age (in years)		0.018*	0.005*	0.026*
18-24	116 (16.8)	60 (51.7)	83 (72.8)	90 (79.6)
25-34	208 (30.2)	100 (48.1)	138 (66.3)	154 (74.0)
35-44	183 (26.6)	71 (38.8)	110 (60.1)	128 (69.9)
45-54	113 (16.4)	37 (32.7)	60 (53.6)	69 (61.1)
55 and above	69 (10.0)	30 (43.5)	35 (50.7)	45 (67.2)
Region of residence		0.065	0.123	0.514
Southern	584 (84.8)	245 (42.0)	355 (61.1)	411 (71.0)
Northern	105 (15.2)	53 (50.5)	71 (67.6)	75 (71.4)
Marital status		0.248	0.072	0.026*
Single	292 (42.8)	138 (47.3)	195 (67.2)	221 (76.5)
Married and living together	343 (50.2)	138 (40.2)	19 (59.4)	23 (71.9)
Married but staying alone	32 (4.7)	14 (43.8)	202 (59.1)	233 (67.9)
Widowed/No longer married	16 (2.3)	5 (31.2)	7 (43.8)	8 (50.0)
Educational status		0.768	0.403	0.035*
Primary	3 (0.3)	1 (50.0)	1 (50.0)	2 (100)
Secondary	61 (8.9)	29 (47.5)	42 (70.0)	50 (84.7)
Tertiary	625 (90.8)	268 (42.9)	383 (61.5)	434 (69.8)
Occupation		0.022*	0.029*	0.019*
Not employed/student	123 (18.5)	66 (53.7)	90 (74.4)	100 (82.6)
Health worker	307 (46.2)	122 (39.7)	185 (60.5)	205 (67.4)
Government employee	127 (19.1)	48 (37.8)	74 (58.3)	90 (70.9)
Private employee	108 (16.3)	54 (50.0)	65 (60.2)	78 (72.2)

*: values significant at p < 0.05; Chi-square test of socio-demographic variables and their choices (willing/unwilling) to the willingness questions.

**Acceptance of COVID-19 vaccine:** of the three survey questions on acceptance to be vaccinated with a hypothetical COVID-19 vaccine, 43.3% (n=298) of the respondents reported that they would accept a vaccine now (if it is available); 62.1% (n=426) reported they would consider taking the vaccines in the future while 486 (71.1%) of the total agreed to accept the vaccine if it is recommended by their healthcare provider. Respondents´ age, occupation, religion and their awareness of any available COVID-19 vaccine were associated with willingness to accept a COVID-19 vaccination now, in the future or when recommended by their health provider. Younger respondents aged 18 to 25 years and 25 to 34 years were more willing compared to older respondents to receive a COVID-19 vaccination during any of the three scenarios (p<0.05 in all). A significantly lower proportion of health care workers (39.7% and 67.4%) and government employees (37.8% and 70.9%) compared to other occupation groups said they would receive a vaccination now or even when recommended by their colleagues respectively (p<0.05). Islamic respondents were significantly more willing to accept the vaccination under any of the three scenarios surveyed compared to their Christian and traditionalist counterparts (p<0.05). Interestingly, respondents who said they were aware of a COVID-19 vaccine soon to be released were reluctant to get vaccination when it does or another time in the future. The study found no evidence of an association between willingness to get vaccinated and the respondents´ age, gender, region of residence, underlying disease and history of ever tested for COVID-19 (Table 1 (suite)).

**Table 1 (suite):** sociodemographic characteristics of the respondents and their willingness to accept COVID-19 vaccines

Variable	Total	Accept vaccination now	Accept vaccination in near future	Accept vaccination if recommended
	**n (%) willing**			
**Religion**		0.022*	0.011*	0.012*
Christianity	632 (92.1)	268 (42.4)	386 (61.4)	441 (70.3)
Islam	47 (6.9)	28 (59.6)	37 (78.7)	41 (87.2)
Traditional religion	7 (1.0)	1 (14.3)	2 (28.6)	3 (42.9)
**Any underlying disease**		0.469	0.384	0.509
Yes#	120 (17.4)	51 (42.5)	72 (60.5)	85 (71.4)
None	569 (82.6)	247 (43.4)	354 (62.4)	401 (71.0)
**Previously tested?**		0.115	0.218	0.156
No	633 (91.9)	269 (42.5)	388 (61.6)	450 (71.7)
Yes	56 (8.1)	29 (51.8)	38 (67.9)	36 (64.3)
**Any family/friend tested?**		0.713	0.056	0.786
No	364 (53.1)	152 (41.8)	215 (59.4)	255 (70.1)
Yes	104 (15.1)	97 (44.7)	149 (68.7)	155 (72.4)
Not really sure	21 (31.7)	47 (45.2)		74 (72.5)
**Aware of any approved COVID-19 vaccine**?		0.017*	0.026*	0.154
No	488 (70.8)	224 (45.9)	313 (64.5)	352 (72.3)
Yes	201 (29.2)	74 (36.8)	113 (56.2)	134 (68.0)

#Participant mentioned hypertension (n=62), diabetes (n=6), asthma (20), others (n=22); *: values significant at p < 0.05; Chi-square test of socio-demographic variables and their choices (willing/unwilling) to the willingness questions

**Reasons for accepting or rejecting hypothetical COVID-19 vaccines:** a total of 477 respondents provided reasons for not accepting the COVID-19 vaccination under any scenario and that generated a total of 677 reasons, while 192 respondents willing to accept the vaccination gave a total of 477 reasons. Respondents willing to accept vaccination had more reasons per respondents for their acceptance compared to those who rejected vaccination who majorly gave one reason for their rejection. Protection of self (31.9%) and protection of one´s family (25.2%) were the main reasons for accepting vaccination. Half of the respondents (i.e. 51.3%) who were unwilling to receive COVID-19 vaccination said they “did not want to be used for an experiment” (Table 2).

**Table 2:** respondents' reasons for rejecting or accepting the COVID-19 vaccine

Unwilling to accept COVID-19 vaccination (N=477)	n (%)	Willing to accept COVID-19 vaccination (N=192)	n (%)
**Frequency of response per participant**			
One	349(73.2)	One	65(33.9)
Two	71(14.9)	Two	31(16.1)
Three	47(9.9)	Three	47(24.5)
Four or more	10(2.1)	Four or more	49(25.5)
**All responses received (N = 676)**		**All responses received (N = 477)**	
I don’t want to be used for an experiment	347(51.3)	Protect myself	152(31.9)
I really don't know why	78 (11.5)	Protect my family	120(25.2)
I don't need the vaccine	74 (10.9)	Stop the outbreak	101(21.2)
Vaccines have side effects	52 (7.7)	Vaccines are safe	51 (10.7)
COVID-19 is not as severe	44 (6.5)	It is a civic duty	26 (5.5)
Vaccines are not safe	43 (6.4)	It is convenient	17 (3.6)
I don't like injections	15 (2.2)	Vaccines have no side effects	8 (1.7)
COVID-19 vaccines are not effective	13 (1.9)	I don't have any reason	2 (0.4)
I have medical reasons	10 (1.5)		

**Factors influencing willingness to be vaccinated:** respondent´s age, religion, test history and awareness of a COVID-19 vaccine influenced their willingness to vaccinate now, in the future and on recommendation. Specifically, older respondents (45 to 54 years) were less likely to accept to be vaccinated for COVID-19 now (aOR= 0.330, 95% CI: 0.141-0.767) and in the future (aOR=0.335, 95% CI: 0.148-0.851) compared to the youngest respondents. Islamic respondents were twice and thrice more likely to accept to be vaccinated in the future (aOR=2.179, 95% CI: 1.012-4.593) and on recommendation of a health provider (aOR=3.251, 95% CI: 1.301-8.093). Also, respondents were twice or thrice more likely to accept to be vaccinated immediately if they had been tested for COVID-19 (aOR=1.912, 95% CI: 1.031-3.546). Lastly, respondents were less likely to accept a COVID-19 vaccine now (aOR=0.637, 95% CI: 0.441-0.920) and in the future (aOR=0.636, 95% CI: 0.440-0.920) if they had been aware of a COVID-19 vaccine soon to be made available (Table 3).

**Table 3:** multivariate logistic regression analysis of respondents' willingness to accept vaccination (3 scenario of unwillingness)

	Accept vaccine now		Accept vaccine in future		Accept vaccine if recommended	
Variables and variable levels	aOR (95% CI)	P	aOR (95% CI)	P	aOR (95% CI)	P
**Gender**						
Female	Ref		Ref		Ref	
Male	1.415 (0.999-2.002)	0.050	1.234 (0.866-1.760)	0.245	1.065 (0.729-1.555)	0.744
**Age**						
18-24	Ref		Ref		Ref	
25-34	0.853 (0.487-1.494)	0.578	0.748 (0.406-1.380)	0.353	1.068 (0.552-2.066)	0.844
35-44	0.471 (0.219-1.010)	0.053	0.494 (0.221-1.105)	0.086	1.038 (0.441-2.446)	0.931
45-54	0.330 (0.142-0.767)	0.010*	0.355 (0.148-0.851)	0.020*	0.680 (0.271-1.702)	0.410
55 and above	0.486 (0.193-1.225)	0.486	0.276 (0.106-0.717)	0.008*	0.938 (0.336-2.620)	0.902
**Region of residence**						
(Southern	Ref		Ref		Ref	
Northern	1.294 (0.817-2.049)	0.272	1.141 (0.704-1.848)	0.593	0.742 (0.286-1.930)	0.358
**Marital status**						
Single	Ref		Ref			
Married and living together	1.383 (0.566-3.382)	0.477	1.058 (0.429-2.608)	0.903	0.742 (0.286-1.930)	0.541
Married but staying alone	1.265 (0.748-2.141)	0.380	1.251 (0.727-2.152)	0.419	0.832 (0.467-1.482)	0.532
Widowed/No longer married	0.702 (0.185-2.659)	0.603	1.035 (0.308-3.475)	0.956	0.449 (0.103-1.551)	0.206
**Education**						
Primary	Ref		Ref		Ref	
Secondary	0.499 (0.028-9.064)	0.639	1.095 (0.058-20.603)	0.952	0.000	0.999
Tertiary	0.707 (0.042-11.830)	0.809	1.256 (0.074-21.373)	0.875	0.000	0.999
**Occupation**						
Unemployed/Student	Ref		Ref		Ref	
Health worker	0.609 (0.336-1.102)	0.101	0.641 (0.333-1.237)	0.185	0.547 (0.270-1.111)	0.095
Government employee	0.608 (0.304-1.217)	0.061	0.633 (0.300-1.335	0.230	0.725 (0.322-1.633)	0.437
Private employee	0.939 (0.472-1.869)	0.858	0.616 (0.293-1.292)	0.200	0.659 (0.0.295-1.475)	0.310
**Religion**						
Christianity	Ref		Ref		Ref	
Islam	1.799 (0.927-3.493)	0.083	2.179 (1.012-4.593)	0.047*	3.251 (1.306-8.093)	0.011*
Traditional religion	0	0.099	0.097 (0.010-0.901)	0.040*	0.208 (0.035-1.250)	0.086
**Any underlying Disease?**						
Yes	Ref		Ref		Ref	
None	0.892 (0.567-1.402)	0.620	0.911 (0.574-1.446)	0.692	0.803 (0.487-1.323)	0.389
**Have you been tested?**						
No	Ref					
Yes	1.912 (1.031-3.546)	0.040*	1.256 (0.655-2.409)	0.493	0.726 (0.383-1.377)	0.327
**Has any family/relative been tested?**						
No	Ref		Ref		Ref	
Yes	1.012 (0.690-1.483)	0.952	1.487 (1.001-2.211)	0.050	1.193 (0.784-1.814)	0.410
Maybe	1.126 (0.706-1.797)	0.618	0.937 (0.585-1.500)	0.054	1.193 (0.707-2.014)	0.509
**Aware of any approved COVID-19vaccine?**						
No	Ref		Ref		Ref	
Yes	0.637 (0.441-0.920)	0.016*	0.636 (0.440-0.920)	0.016*	0.857 (0.579-1.270)	0.442

**Figure 1:**
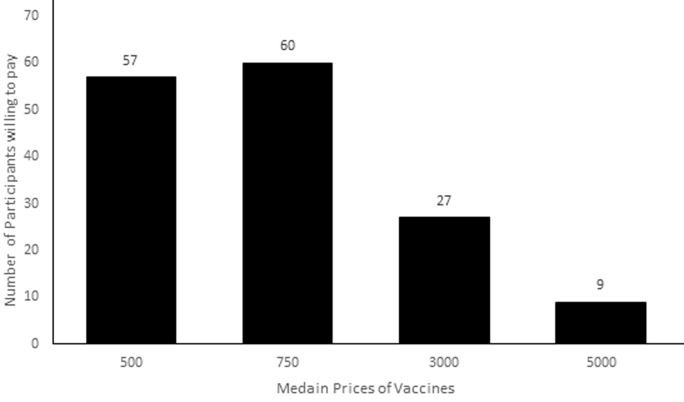
distribution of respondents' and their willingness to pay (WTP) for COVID-19 vaccination if it is not free

**Willingness to pay for the vaccine:** a total of 157 respondents (representing 81.7% of those who were willing to accept the vaccination free of charge) reported that they were willing to pay some money if the vaccine was not free. The median range of WTP was between 500-1000N (US $ 1.2 -2.5). The distribution of respondents according to how much WTP revealed that as price range increased, the number of respondents´ WTP increased till the maximum amount range. Exactly 18.9% said their WTP amount was less than US $1.2, 36.4% said WTP is between US $1.2-2.5, 39.2% said WTP is between US$ 2.5-12.0. Only 5.6% selected the maximum WTP of greater than US$ 12.0 for a shot of the vaccine (Figure 1).

## Discussion

Presently, a few candidate vaccines against COVID-19 for use in human have reached advanced stages and are ready for mass distribution after passing through the final stages of clinical trial. The first to be announced was that of Pfizer/BioNTech with over 90% effectiveness. That was followed by the one by Moderna, also with effectiveness above 90%, and finally that of AstraZeneca [9]. The import of this news is that a vaccine with considerable protection against the COVID-19 virus which also safe will be available for public use soon. Thus, this study was conducted to determine the reception of the Nigerian populace to a hypothetical COVID-19 vaccine, as well as the amount that they would be willing to pay, if they agree to pay, in the event that the vaccine is not available for free administration.

**Sociodemographic characteristics:** the distribution of the sociodemographic characteristics of the respondents in this study showed that majority were middle aged, having ages between 25 and 44 years. There was an almost equal distribution of other characteristics such as gender and marital status. Healthcare workers were about half of the entire study sample. The bias for healthcare professionals in the occupation of the respondents was done with the realization that they have chances of being exposed to the disease by virtue of their contact with patients. Nigeria has not conducted an official census for over a decade to establish an objective statistic on the sociodemographic characteristics of the citizens. However, data from the country´s National Bureau of Statistics showed that majority of the citizens were aged below 44 years while there were more females than males [10]. Currently, there is a disproportionate number of healthcare professionals to serve the Nigerian populace, as it is in most developing country. The World Health Organization (WHO) reported recently that there are about 20.1 physicians, nurses and midwives per 10,000 population [11]. It was reported that, as at 2016, the density of pharmacists per 10,000 population in Nigeria was 0.66 [12]. It is therefore important that the views of healthcare professionals on their willingness to accept the COVID-19 vaccine are captured.

**Acceptance of COVID-19 vaccine:** respondents in this survey reacted to the three scenario questions on willingness to accept vaccination differently. The acceptance of a COVID-19 vaccination presently if made available was very low at 43%. A much higher percent, 62% of the respondents said they would consider vaccination in the future. This may suggest that time might be an influence in the decision making for acceptance to be vaccinated. The pattern of these responses cut across the entire population irrespective of sociodemographic factors, except for age and occupation. The findings of this study were similar to those of a study by Reiter *et al*. in the United States where 69% of their respondents indicated a positive willingness to accept a vaccine against COVID-19 [3]. Nonetheless, the findings of this study and that of Reiter *et al*. were low when compared to those of Harapan *et al*. which was conducted in Indonesia [13]. In a global study by Lazarus *et al*. conducted 5 months earlier, a subset of the respondents were Nigerians and the study reported a 65% acceptance to be vaccinated [14]. This might suggest increased hesitancy during the period of study as the pandemic continually worsened. The study also revealed variations in the acceptability to COVID-19 vaccines in different countries with findings showing highest acceptance of 88.62% in China least acceptance of 54.85% in Russia. However, more respondents in our survey were willing to receive the vaccination if their healthcare provider recommended it and this has also been reported in another study on COVID-19 vaccination 2. This opens a huge possibility to drive mass vaccination in a country like Nigeria where mis-information of the COVID-19 virus is rampant and government is struggling to counter such news. While healthcare providers and front-line workers have been suggested to be first in line to receive vaccination, it is important to get them to be committed in driving the mass vaccination campaign as the public still retains some trust in them. Provider recommendation is key in influencing vaccine acceptance and their efforts to increase COVID-19 vaccine uptake needs to be strategically considered. Vaccine hesitancy among healthcare provider is common and had been previously reported in literature regarding other viral infections. Whereas about 60% of the physicians in a French study were willing to accept A/H1N1 pandemic vaccination, [15] only 24% of health workers were willing to accept pre-pandemic H5N1 vaccine in Hong Kong [16]. In the same Hong Kong, about the same proportion were willing to accept an influenza A (H1N1) vaccine [17]. This present study did not investigate vaccine acceptance among healthcare providers but its findings suggest its possible exploration so as to evaluate providers´ willingness to get vaccinated and to also recommend vaccination to their clients.

The main reason for respondents´ unwillingness to be vaccinated with any COVID-19 vaccine was not “to be used as an experiment”. This reason had earlier been fueled by past experiences and culture-religious sentiments. During the early months of the pandemic, misinformation on the 5G technology and the Gates Foundation spread across the continent and it possibly has left a scar too difficult to be healed. Building public trust and confidence should be priority as the continent waits for the vaccine. Government and non-governmental agencies must act as trusted sources of information on safety and effectiveness of any vaccine to be procured. There should also be some engagement with the religious and traditional leaders of the community as their role in correct information dissemination is very important. Respondents´ age, religious inclination, previous test history and awareness of a potential vaccine influenced their willingness to accept vaccination. Older patients were less willing to accept COVID-19 vaccination if available now or in the future. This finding was concerning as this same group are said to be at high risk of getting infected and would benefit most from the vaccination. In the Indonesian study, a similar result was also obtained, where retired workers were most unwilling to get vaccinated for COVID-19 [13]. Poor access to information and consequent inadequate knowledge of the disease might be reasons for such a finding as most information on COVID-19 is on the social media and not traditional media. Christians were as much as twice to thrice more unwilling to get vaccinated. This finding has not been reported earlier in literature. An earlier study conducted in the United States, reported no effect of religion on acceptance of the COVID-19 vaccine [18]. In contrast, Africa is a continent where religion plays a role in decision making including in health care. As earlier mentioned, some Christian leaders significantly questioned the existence of the virus, the safety of any vaccine to be made, and the politicization of the 5G network with the COVID-19 pandemic. These actions might still have their effects as seen in the study findings and could further worsen the continental and global drive to get as much people vaccinated. A careful balance in re-educating the public without raising religious issues that might further worsen vaccine hesitancy should be explored. Awareness of a potential vaccine did not improve willingness to accept it. This finding does raise some concern and should be further explored. While it is commonly known that awareness of a thing might not change one´s practice towards such, the need for global vaccination for COVID-19 is so important for the life of many. Information on the vaccine effectiveness and safety should form the majority of information being released as the wait for the vaccine continues to help boost public confidence in accepting the vaccine. It has also been suggested that the inclusion of a COVID-19 vaccine in the routine immunization schedule may improve its uptake in Africa [19].

**Willingness to pay for the vaccine:** not many of the respondents were willing to make out of-pocket payment to access the vaccine, if it eventually was not made available for free. However, majority of those who agreed to pay for the vaccines were those that had already indicated positive acceptance for the vaccination if it was to be provided free of any payment. Yet, the amount that they offered to pay for the vaccine was low, with almost all the respondents not agreeing to pay above US$12 dollars. An increase in the cost assigned to the vaccine led to a decrease in the proportion of the respondents that were willing to pay for the vaccine. Only a handful of studies have assessed citizens´ WTP for a vaccine against COVID-19. Earlier in May 2020, a study was conducted by Berghea *et al*. in Romania to determine the WTP for a hypothetical COVID-19 vaccine by their respondents [20]. Although they did not report the composite positive WTP proportion of the study sample of 203 respondents, they reported that the amount that their respondents were willing to pay to be vaccinated against COVID-19 was 20-200 Euro. The range is of course far above the ones reported in the present study. They added that the income of the respondents was the main determinant of their WTP amounts. Two months after the Romanian study, a contingent valuation study in Chile reported that the WTP for a hypothetical vaccine against COVID-19 was high, with about 90% of the 566 respondents expressing positive WTP [21]. The average amount that they obtained from the respondents was US$184.72 for the vaccine. Also, a recently published study that was conducted in Indonesia by Harapan *et al*. reported that about 80% of their 1359 respondents were willing to pay for a COVID-19 vaccine [22]. The average amount that they obtained from the respondents was US$ 57.20 (95%CI: US$ 54.56 - US$ 59.85). The amount to be paid by the respondents is also higher than the one that was obtained from the participants of this study. The possible reasons for a lower amount by the respondents of the present study compared to others could be that those other countries have better economic indices compared to Nigeria. In addition, those countries have higher prevalence of COVID-19 infection than Nigeria, although they are reported to have tested a larger number of people.

## Conclusion

There is a low acceptance in the Nigerian community for a COVID-19 vaccine whenever it is available, but the acceptance was significantly higher if it is recommended by healthcare provider. The willingness to accept vaccination was influenced by age and religion of the respondents, as well as their awareness of a potential vaccine in production. Many of the respondents, willing to be vaccination free of charge, indicated a positive WTP for the vaccine, though of a small value. Future strategies in improving vaccination uptake could focus on these influencing factors.

### What is known about this topic

- COVID-19 vaccine uptake hesitancy is a global issue influenced mostly by mass dis-information;
- Low vaccine uptake would hinder the eradication of the virus and slow the development of herd immunity.

### What this study adds

- COVID-19 vaccine hesitancy is very much prevalent among Nigerians and is influenced by age and religion;
- Willingness to accept COVID-19 vaccines was greatly improved if health care providers were to recommend the vaccination.
